# Body-size trends of the extinct giant shark *Carcharocles megalodon*: a deep-time perspective on marine apex predators

**DOI:** 10.1017/pab.2015.16

**Published:** 2015-06

**Authors:** Catalina Pimiento, Meghan A. Balk

**Affiliations:** Florida Museum of Natural History, University of Florida, Gainesville, Florida 32611, U.S.A.; Department of Biology, University of Florida, Gainesville, Florida, U.S.A.and Smithsonian Tropical Research Institute, Box 2072, Balboa, Panama. E-mail: pimientoc@ufl.edu; University of New Mexico, Albuquerque, New Mexico 87131, U.S.A.

## Abstract

The extinct shark *Carcharocles megalodon* is one of the largest marine apex predators ever to exist. Nonetheless, little is known about its body-size variations through time and space. Here, we studied the body-size trends of *C. megalodon* through its temporal and geographic range to better understand its ecology and evolution. Given that this species was the last of the megatooth lineage, a group of species that shows a purported size increase through time, we hypothesized that *C. megalodon* also displayed this trend, increasing in size over time and reaching its largest size prior to extinction. We found that *C. megalodon* body-size distribution was left-skewed (suggesting a long-term selective pressure favoring larger individuals), and presented significant geographic variation (possibly as a result of the heterogeneous ecological constraints of this cosmopolitan species) over geologic time. Finally, we found that stasis was the general mode of size evolution of *C. megalodon* (i.e., no net changes over time), contrasting with the trends of the megatooth lineage and our hypothesis. Given that *C. megalodon* is a relatively long-lived species with a widely distributed fossil record, we further used this study system to provide a deep-time perspective to the understanding of the body-size trends of marine apex predators. For instance, our results suggest that (1) a selective pressure in predatory sharks for consuming a broader range of prey may favor larger individuals and produce left-skewed distributions on a geologic time scale; (2) body-size variations in cosmopolitan apex marine predators may depend on their interactions with geographically discrete communities; and (3) the inherent characteristics of shark species can produce stable sizes over geologic time, regardless of the size trends of their lineages.

## Introduction

The extinct megatooth shark *Carcharocles megalodon* is the largest shark ever to exist (Gottfried et al. 1996). From its tooth size and morphology, it was inferred to have been an apex predator that reached up to ~18 m of total length (TL) (Gottfried et al. 1996; Pimiento et al. 2010; Pimiento et al. 2013a). Furthermore, given the nearly global distribution of its fossil record, *C. megalodon* is considered to have been a cosmopolitan species that lived from ca. 15.9 Ma (middle Miocene) to ca. 2.6 Ma (Pliocene/Pleistocene boundary) (Applegate and Espinosa-Arrubarrena 1996; Gottfried et al. 1996; Purdy 1996; Purdy et al. 2001; Cappetta 2012; Pimiento and Clements 2014).

Apex predators are animals with no predatory pressures. Usually they are large-bodied vertebrates that can move over large areas, thus interacting with different communities. Most importantly, apex predators are pivotal in maintaining ecosystem stability, and their elimination can produce cascading effects throughout entire food webs (Myers et al. 2007; Terborgh et al. 2010; Estes et al. 2011). Accordingly, the extinction of *C. megalodon* potentially affected the structure and function of ancient ecosystems (Pimiento and Clements 2014). The causes of its extinction are still unknown.

The phylogenetic relationships of *C. megalodon* have mainly been studied on the basis of its relatedness to the great white shark, *Carcharodon carcharias* (e.g., Long and Waggoner 1996; Martin 1996). To our knowledge, no phylogenies for this species have ever taken into consideration all its ancestors. Thus, the taxonomy of *C. megalodon* has long been debated, with a number of possible interpretations. For instance, some authors place it in the genus *Carcharodon* (family Lamnidae) (e.g., Applegate and Espinosa-Arrubarrena 1996; Gottfried et al. 1996; Purdy 1996), whereas others place it in the genus *Carcharocles* (Family Otodontidae) (e.g., Ward and Bonavia 2001; Nyberg et al. 2006; Ehret et al. 2009; Ehret 2010; Pimiento et al. 2010; Cappetta 2012). Using the most recent morphological evidence (e.g., Nyberg et al. 2006; Ehret et al. 2009), we follow the second interpretation.

**Figure 1:**
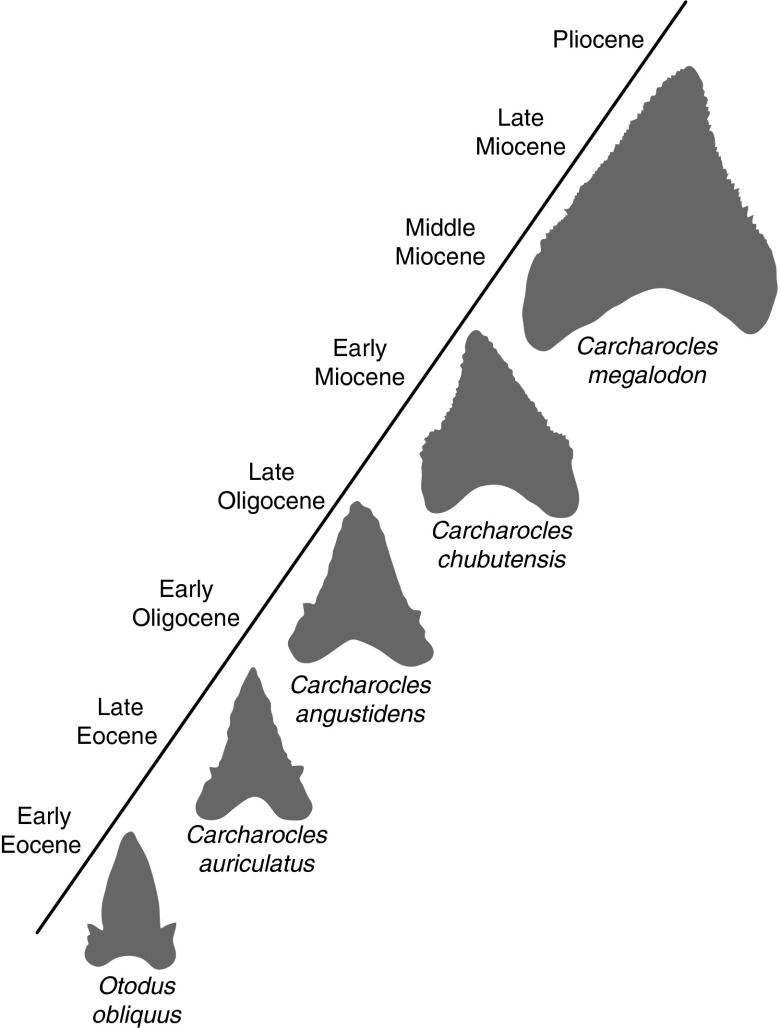
Schematic representation of the changes in tooth morphology within the megatooth lineage: cusplet loss, broadening of tooth crowns, and size increase. Scheme based on the work of Ehret (2010).

Regardless of its taxonomic assignment, it is widely accepted that *C. megalodon* is the largest member of the megatooth lineage, an extinct group of large predatory sharks. It has been proposed that the megatooth sharks comprise a series of chronospecies (i.e., a group of species that evolve via anagenesis and that gradually replace each other in a evolutionary scale [Benton and Pearson 2001]) that are distinguished from each other in the fossil record by the morphological changes of their teeth (Ward and Bonavia 2001). These changes include the loss of lateral cusplets (Ward and Bonavia 2001; Ehret 2010; Pimiento et al. 2010; Pimiento et al. 2013b); broadening of tooth crowns; and, of most relevance to this study, size increase through geologic time (Ehret 2010). Because tooth size has been demonstrated to be a good proxy of body size in lamnoid sharks (Gottfried et al. 1996; Shimada 2003; Pimiento et al. 2010), we can infer that the observed chronoclinal tooth size trend of the megatooth linage (Fig. 1) translates into a macroevolutionary body-size increase over geologic time.

Body size has long been of interest to scientists, not only because it is a relatively easy trait to quantify in both living and fossil organisms (Peters 1983; Maurer et al. 1992; Kingsolver and Pfennig 2004; Smith et al. 2008), but also because it correlates with many ecological and evolutionary patterns (Peters 1983; Calder 1996; Smith et al. 2008). For example, body-size distributions are an important component of community structure and thus are often studied to infer selection pressures (Peters 1983; Werner and Gilliam 1984; Bell et al. 2006). Furthermore, body size is highly correlated with geographic distribution, making it the most common and repeatable relationship studied in macroecology (Lyons and Smith 2010).

Body size has important implications for a species’ ecology. Many clades have a log-skewed (right-skewed on logarithmic axes) body-size distribution pattern, where the majority of species are small and a few are large (Kozlowski and Gawelczyk 2002; O’Gorman and Hone 2013). This pattern has been demonstrated in mammals, birds, reptiles, amphibians, and fish, but not in dinosaurs (left-skewed) or snakes (not skewed) (Boback and Guyer 2003; Lyons and Smith 2010; O’Gorman and Hone 2013). Moreover, body-size patterns are driven by clade- or region-specific mechanisms, which produce both positive and negative correlations between body size and latitude (Cushman et al. 1993; Atkinson 1994). It has also been argued that body-size distributions are invariant along latitudinal gradients (Roy et al. 2000). To our knowledge, there have been no studies investigating body-size trends (either body-size distributions or body-size geographic patterns) at the species level of any marine apex predator over a geologic time scale.

Little is known about the body-size trends of the extinct apex predatory shark *C. megalodon* over geologic time. Because body size predictably scales with many aspects of species’ biology, here we study body-size trends of *C. megalodon* across time and space as a means to better understand the ecology and evolution of this species. Given that *C. megalodon* was the largest of a lineage with a purported body-size increase over time, we hypothesize that this species increased in size through time, reaching its largest size prior to extinction. In order to reach our research objectives and test our hypothesis, we estimated the body size of individuals from a large sample across regions and time periods, compared trends through the species’ temporal and geographic range, and tested its general mode of size evolution. Our results provide novel information on the macroecological patterns of this extinct giant shark. Moreover, because *C. megalodon* is a long-lived species (~14 Myr) with a widely distributed fossil record, it represents an ideal study system to provide a deep-time perspective to the understanding of body-size trends of marine apex predators.

## Methods

### 

#### Museum Collections Survey

We did an online search of natural history museums throughout the world that house specimens encompassing the species’ known temporal and latitudinal range. In order to identify which of these museums contain sufficient material, we explored their databases and/or requested a list of specimens. As a result of this process, we visited the following museum collections: the British Museum of History Museum (NHM); Museo Argentino de Ciencias Naturales “Bernardino Rivadavia” (MACN); Museo de La Plata (UNLP); Museo de Historia Natural de la Universidad de San Marcos, Lima (UNMSM); Museo Nacional de Historia Natural de Chile (MNHN); Florida Museum of Natural History (FLMNH); Natural History Museum of Los Angeles County (LACM); San Diego Natural History Museum (SDNHM); University of California Museum of Paleontology (UCMP); and Smithsonian Institution National Museum of Natural History (USNM). After examining their specimens for signs of abrasion (as an indicator of redeposition; e.g., Boessenecker et al. 2014), we selected only well-preserved, relatively complete specimens with adequate stratigraphic information for inclusion in our study.

#### Tooth Measurements

**Figure 2:**
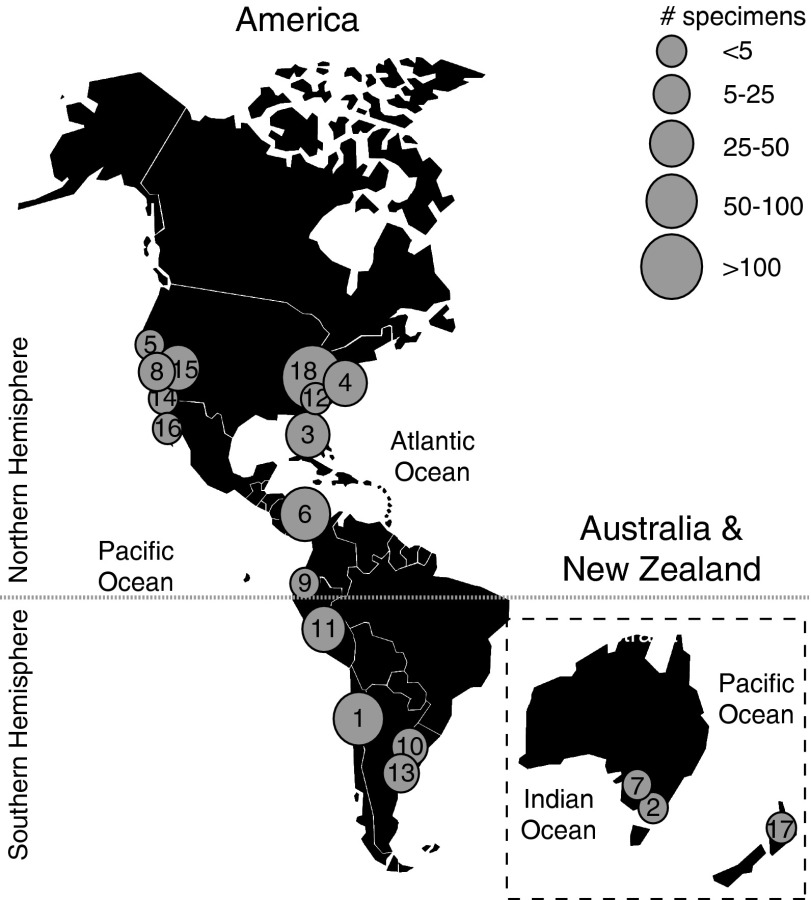
Geographic locations of *Carcharocles megalodon* collections included in this study. 1. Bahia Inglesa Fm., Mina Fosforita, late Miocene (MNHN). 2. Basal Black Rock Sandstone Fm., Beaumaris, Pliocene; Batesford Fm., Batesford, Middle Miocene; Muddy Creek Fm., Hamilton, late Miocene (NHM). 3. Bone Valley Fm., Payne Creek Mine, Fort Green Mine SW, North Palmetto Mine, Achan Mine, Palmetto Mine (Agrico) and Chicora Mine (FLMNH); Tamiami Fm., East Coast Aggregates, Pliocene (FLMNH). 4. Calvert Fm., Parkers Creek and Scientists Cliff, middle Miocene localities (USNM and LACM). 5. Capistrano Fm., Laguna Hill and Antigua; Purisima Fm., Steamer’s Lane, late Miocene (LACM, UCMP and SDNHM). 6. Chucunaque Fm., late Miocene; Gatun Fm., YPA017, YPA021 and YPA032, late Miocene and YPA033, middle Miocene (FLMNH). 7. Loxton Sand Fm. Sunlands Pumping Station, Pliocene (NHM). 8. Monterey Fm., Altamira, El Toro and Leisure World, middle Miocene; San Mateo Fm., Lawrence Canyon, late Miocene and Lawrence Canyon upper gravel unit, Pliocene; Topanga Fm., Cook’s Corner, middle Miocene (LACM and SDNHM). 9. Onzole Fm., Punta la Gorda and Punta la Colorada, Pliocene (NHM). 10. Paraná Fm., late Miocene (MACN and UNLP). 11. Pisco Fm., Cerro Colorado, middle Miocene; Montemar, Cerro Los Quesos, Cerro La Bruja, Yesera Amara, Ocucaje, Agua de las Lomas, late Miocene (UNMSM). 12. Pungo River Fm., Middle Miocene (USNM). 13. Punta del Diablo Fm., late Miocene (UNLP). 14. Rosarito Beach Fm., Mesa los Indios, middle Miocene (SDNHM). 15. Temblor Fm., Shark Tooth Hill, middle Miocene (LACM and UCMP). 16. Tirabuzon Fm., Baja, Pliocene; Ysidro Fm., Santa Rita, middle Miocene (LACM and SDNHM). 17. Wanganui, Wellington, Pliocene (NHM). 18. Yorktown Fm., Pliocene (LACM and USNM).

We measured tooth crown height (CH) and width (CW) of a total of 544 *C. megalodon* specimens from 32 localities, 26 formations, and nine countries (Fig. 2). Another 51 specimens were measured; however, they either showed signs of redeposition or lacked sufficient stratigraphic information to be included in our analyses. These include 30 teeth from the Red Crag Formation (U.K.) that were clearly eroded, and 21 specimens from the Middle Globigerina Limestone (Malta) that did not have accurate stratigraphic information. These teeth are all deposited in the NHM collection.

#### Body-Size Estimations

We estimated the total length (TL) of *C. megalodon* teeth measured following the methods described in Pimiento et al. (2010), where the tooth CH is used to calculate TL based on the regressions from Shimada (2003) on the great white shark (*Carcharodon carcharias*), which is considered a modern analogue of *C. megalodon*. Accordingly, every tooth position in the jaw corresponds to a regression equation that calculates body size. As in Pimiento et al. (2010), we assigned a range of plausible positions to each tooth and estimated TL of every specimen by calculating it from the average among the different positions where every tooth could have belonged.

We then created a matrix of data (available in online supplemental materials) consisting of specimen number, CH, CW, tooth position, TL, geologic age (maximum, minimum and median), epoch, stage, formation, locality, stratigraphic level, country, ocean, latitude and collection. Our data collection covers a large portion of *C. megalodon*’s geographic distribution range (Pacific, Atlantic, and Indian oceans; Northern and Southern Hemispheres). Despite these efforts, we were not able to obtain samples from northern Europe, Asia, or southern Africa, where there are known *C. megalodon* records. Nonetheless, our matrix represents the most comprehensive data set of body-size estimations for this species and, of most relevance for this work, includes all body-size ranges and hence, life stages. We did not exclude any tooth size, as we are not interested in maximum length, but in quantifying overall patterns of body size including all life stages and habitats.

#### Geological Age Assessment

For each specimen studied, we examined the accompanying label and used collection databases to verify the age assignment. Additionally, we studied a number of supplementary references that further documented or refined the age of the localities from which the specimens were recovered. This process was aided by using the Paleobiology Database (http://paleobiodb.org).

#### General Statistical Comparisons

In order to assess *C. megalodon* body-size trends through time, we calculated the moments (minimum [Min] and maximum [Max] values, mean, mode, skewness, and kurtosis) of the distribution of the TL data. We also divided the data into three time slices based on the age range of the specimens studied (middle Miocene, late Miocene, and Pliocene), following the geologic time scale of Gradstein et al. (2012). We did not subdivide Pliocene into early and late so as to maintain a relatively equitable time span for each slice. Finally, we calculated the moments of the distribution of TL for each time period and made pairwise comparisons of all distributions, using Kolmogorov-Smirnov (KS) tests.

#### Geographic Statistical Comparisons

In order to assess how trends in body size of *C. megalodon* vary across space, we plotted TL by absolute latitude, hemisphere, and ocean. Furthermore, we calculated the linear regression between body size and latitude, as well as compared body size by hemisphere and by ocean, using a Welch two-sample *t*-test and a Tukey test, respectively. Finally, we repeated the comparisons for each time slice. All analyses in this study were made using the statistical software R (R Development Core Team 2012).

#### Evolutionary Models

To test our hypothesis (H1=*Carcharocles megalodon* increased in size through time, reaching its largest size prior to extinction) we used the methods of Hunt (2006, 2008) and Hunt and Carrano (2010). We tested three common models of trait evolution: random walk (UWR), where evolutionary increments are independent and equally likely to increase or decrease; directional evolution (GWR), which features a trend of increasing (or decreasing) trait values over time; and stasis, with trajectories that show fluctuations around a steady mean. We used the R package *paleoTS* (Hunt 2008) to fit these models to our time series of body sizes. This package uses maximum-likelihood estimation to fit these models and the small-sample-size Akaike Information Criterion (AICc) as a measure of model support (Hunt and Carrano 2010). Furthermore, it aids the interpretation of AICc scores by converting them to Akaike weights, which are the proportional support that each model receives.

Our general statistics and geographic analyses over time used three time slices: middle Miocene, late Miocene, and Pliocene. However, for our evolutionary models we used the total number of bins that resulted from estimating the mean age of each sample. For each resulting bin, we calculated the mean, variance, and sample size of the TL data, which formed the basis for the time-series analysis in *paleoTS* (available in online supplemental materials).

#### Supplementary Analyses

Megatooth sharks have diagnathic heterodonty (i.e., differences in the tooth morphology of the upper and lower dentition) (Purdy et al. 2001). Moreover, antero-posteriorly through the jaw, there is a slight initial tooth-size increase followed by a progressive decrease that continues to the last tooth. Because of this tooth-size variability within individuals, we calculated TL of each specimen based on a position-specific regression equation and drew our analyses on the basis of such estimations. Nonetheless, it could be argued that this approach warrants some caution, as TL estimations were based in a modern analogue (*C. carcharias*). To counteract this issue, we repeated all of our analyses using the raw tooth size data (available in online supplemental materials) and contrasted them with our main results using TL. Our conclusions are still based on the results obtained from the analyses data, as they represent a more robust estimation of the body size of *C. megalodon*.

## Results and Discussion

### Ecology

#### General Body-Size Patterns

**Figure 3:**
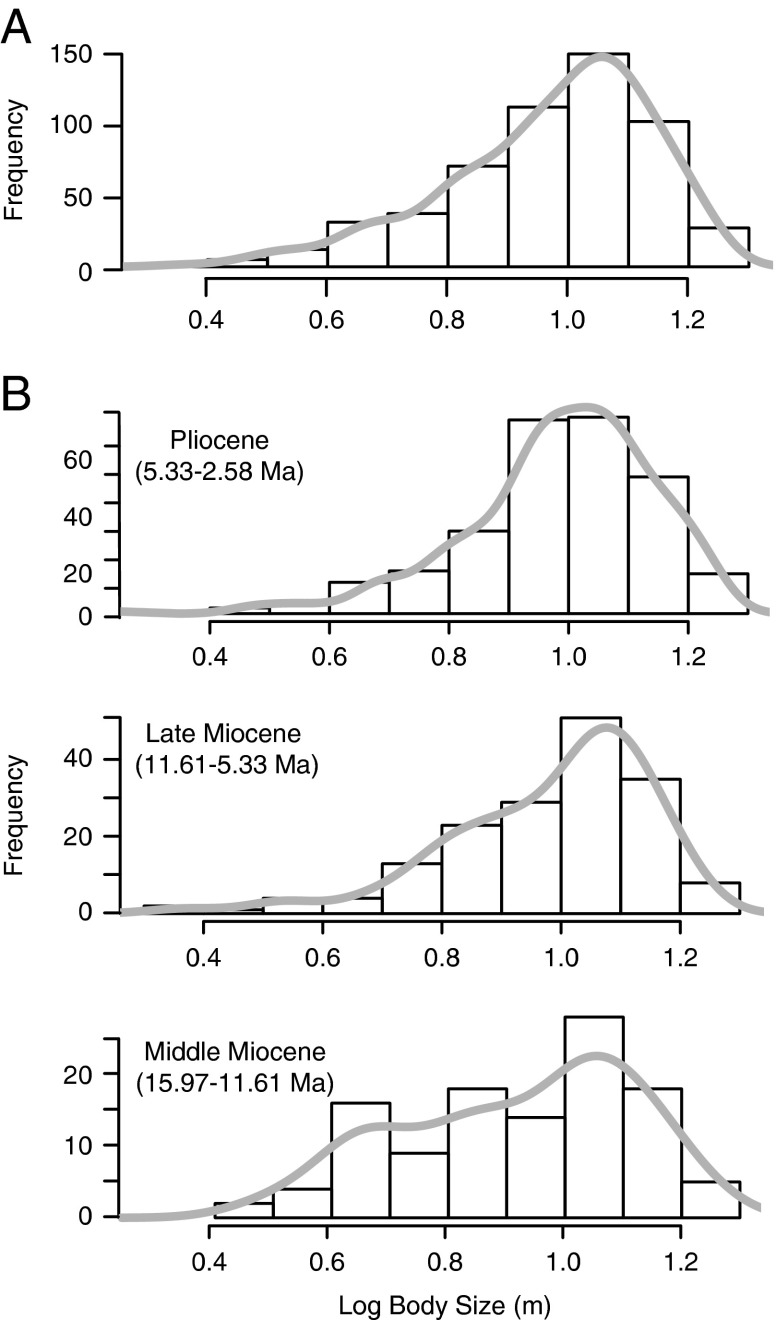
*Carcharocles megalodon* body-size distributions (note log10 scale). The density curve is in gray. A, General body-size distribution. B, Body-size distributions through time.

Total Length (TL) estimates for *Carcharocles megalodon* range from 2.20 to 17.90 m (mean=10.02 m, mode=10.54 m) (Table 1). The distribution of *C. megalodon* body sizes was left-skewed on a log scale (Table 1, Fig. 3A), with larger individuals found more frequently than smaller individuals. Above the species level, body-size distributions are usually right-skewed (Kozlowski and Gawelczyk 2002; O’Gorman and Hone 2013). At narrower taxonomic levels, species’ body sizes are influenced by their unique physiological constraints, ecological relationships, and selective pressures (e.g., McClain et al. 2015). These sets of characteristics result in species having sizes relatively close to their optimum, which in turn shapes their distribution of body-size frequencies (Kozlowski and Gawelczyk 2002).

**Table 1:** Descriptive statistics of *Carcharocles megalodon* body size (m) through time. Significant values in bold. Codes: P=Pliocene (5.33–2.58 Ma), LM=late Miocene (11.61–5.33 Ma), MM=middle Miocene (15.97–11.61 Ma).

	*n*	Min	Max	Mean	Mean (log 10)	Mode	Mode (log 10)	Skew (log 10)	Kurtosis (log 10)	*p*-value (K.S.)
All	544	2.20	17.9	10.02	0.97	10.54	1.02	−0.84	0.43	
P	260	2.92	17.68	10.29	0.99	10.18	1.01	−0.79	0.69	0.58
LM	170	2.20	17.00	10.22	0.98	11.59	1.06	−1.13	1.37	**0.02**
MM	114	2.81	17.90	9.12	0.92	9.32	0.97	−0.37	−0.93	

Optimum size is the size at which there is no ecological advantage to evolving larger or smaller size, and has often been defined as the most frequent size found across a broad scale (Maurer et al. 1992; Brown et al. 1993). The most frequent TL value of *C. megalodon* in a geologic time scale is 10.54 m (mode in Table 1, peak in Fig. 3A). However, it is noteworthy that the optimum size of a species can vary across populations and ontogeny, and can also be taphonomically biased in the fossil record. Regardless, our broad scale results show a higher frequency of larger individuals (left-skewed distribution) and a modal value at 10.54 m that may have shaped this trend.

When comparing *C. megalodon* body-size patterns throughout time (Fig. 3B), we obtained similar moments for each time slice studied (Table 1), with the middle Miocene slice showing a significantly different distribution, lower mode, and less negative skewness relative to the general trend (Table 1). Despite these differences, a left-skewed body-size distribution and a mode around 10.54 m (between 9.32 and 11.59 m) were maintained through time. All these trends are supported by the raw data (Supplementary Table S1, Supplementary Fig. S1).

#### Geographic Trends of Body Size

**Figure 4:**
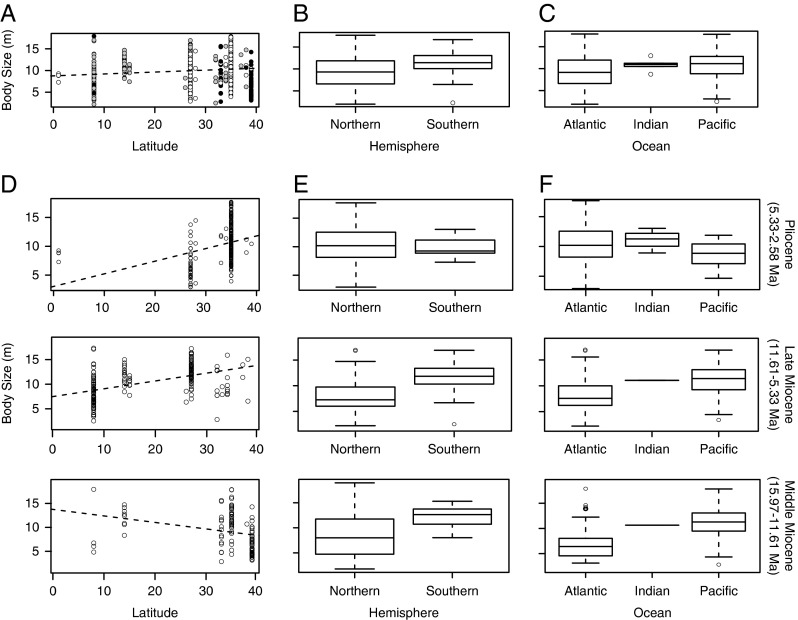
Geographic trends in *Carcharocles megalodon* body size. A, Body size by latitude. The dashed line represents best-fit linear regression model. Black dots represent the middle Miocene (MM) samples, gray dots the late Miocene (LM) samples, and white dots the Pliocene (P) samples. B, Boxplot showing body size by hemisphere. C, Boxplot showing body size by ocean. D, Body size by absolute latitude through time. E, Boxplots showing body size by hemisphere through time. F, Boxplots showing body size by oceanic region through time.

No correlation (*R* ^2^=0.01) was found between TL estimates and absolute latitude (Table 2, Fig. 4A), suggesting that body size did not vary systematically along a latitudinal gradient. Of note, midlatitudes lack fossil occurrences, lower-latitude fossil occurrences are all from the Pliocene (white dots), and higher latitudes are dominated by middle Miocene fossil occurrences (black dots) (Fig. 4A). Whether these patterns are biological or due to sampling bias requires further investigation. Consequently, our geographic distribution results must be interpreted with caution, as they might be influenced by our sampling and/or the availability of outcrops in certain areas and subsequent deposition in major collections (e.g., Uhen and Pyenson 2007).

**Table 2:** Statistical comparisons of *Carcharocles megalodon* body size (m) trends through time across space. Significant values in bold. P=Pliocene (5.33–2.58 Ma), LM=late Miocene (11.61–5.33 Ma), MM=middle Miocene (15.97–11.61 Ma).

	Latitude	Hemisphere		Ocean					
		North-South		Atlantic-Indian		Indian-Pacific		Atlantic-Pacific	
	*R* ^2^	*p*-value	*t*	*p*-value	*t*	*p*-value	*t*	*p*-value	*t*
All	0.01	**<0.01**	−7.17	0.50	1.07	1.00	−0.09	**<0.01**	4.47
P	0.11	0.53	0.65	0.88	0.47	0.38	−1.30	0.22	1.65
LM	0.19	**<0.01**	−8.11	0.64	0.87	1.00	−0.02	**<0.01**	5.04
MM	0.07	**<0.01**	−3.95	0.48	1.12	0.99	0.14	**<0.01**	6.73

Significant differences were found between *C. megalodon* body sizes from the Northern Hemisphere relative to the Southern Hemisphere (Table 2). Notably, the Southern Hemisphere has a larger mean body size (Fig. 4B) (Northern *n*=426, mean=9.58 m, 78.30% of total sample; Southern *n*=118, mean=11.62 m, 21.69% of the total sample). Similarly, significant differences were found between samples from the Atlantic and Pacific oceans, with the Pacific having a larger mean value (Pacific *n*=188, mean=10.90 m, 34.55% of the total sample; Atlantic *n*=350, mean=9.53 m, 64.33%). No significant differences were found between *C. megalodon* body sizes from the Indian Ocean relative to the Atlantic or the Pacific (Table 2, Fig. 4C); however, the low sample size of the Indian Ocean (Indian *n*=6, mean=11.03 m, 1.10% of the total sample) severely limits the statistical power.

The differences in mean sizes across hemispheres and oceans could be due to both environmental (e.g., water depth, ocean currents, resource availability, productivity) and biological (e.g., sexual segregation, habitat use, home range) reasons. On the other hand, it could also be due to sampling and taphonomic biases. For instance, the larger mean size found in the Southern Hemisphere could be the result of a lack of systematic collecting efforts, as most of the southern samples are from the Bahia Formation (Mina Fosforita, Chile, #1 in Fig. 2); these come from illegal confiscations and are biased toward larger teeth (R. Otero personal communication 2013). Similarly, Atlantic specimens come mostly from high latitudes. Even though *C. megalodon* is well known from tropical Atlantic and Caribbean localities (see Pimiento et al. 2013a for a review), large natural history collections from the tropics are lacking, and our samples from the Caribbean included only one collection (Gatun Formation, Panama, #6 in Fig. 2).

In spite of our sampling limitations, we were able to collect a relatively large number of specimens (544) from a broad time range (~14 Myr). Collectively, these specimens suggest that *C. megalodon* body size differs significantly between hemispheres and among ocean basins, but not across a latitudinal gradient. This body-size pattern across space reflects the widespread distribution of *C. megalodon*, which may be a result of its geographically structured populations facing diverse ecological constraints (hence the differences between hemispheres and oceans), even though the species had a cosmopolitan range (hence the lack of a latitudinal gradient).

Similar to the overall pattern, there was no correlation between body size and absolute latitude within any time period. The middle Miocene was particularly similar to the overall relationship (Table 2, Fig. 4D). Also, *C. megalodon* was significantly larger in the Southern Hemisphere and in the Pacific Ocean during the middle and late Miocene (Table 2, Fig. 4E,F). Even when in the Pliocene *C. megalodon* appeared to have slightly larger sizes in the Northern Hemisphere and in the Atlantic Ocean, these differences were not significant (Table 2).

The raw data support each of these trends (Supplementary Table S2, Supplementary Fig. S2), with the Southern Hemisphere having significantly larger tooth sizes throughout all time periods. Although the Indian Ocean data reveal significantly larger tooth sizes both in the total sample and in the Pliocene, this disparity lacks statistical power given the small sample size of the Indian Ocean (*n*=6, 1.10% of the total sample). Nevertheless, taken together, our results suggest that the differences in *C. megalodon* body size across space are maintained throughout time.

### Evolution

#### Evolutionary Body-Size Mode

**Figure 5:**
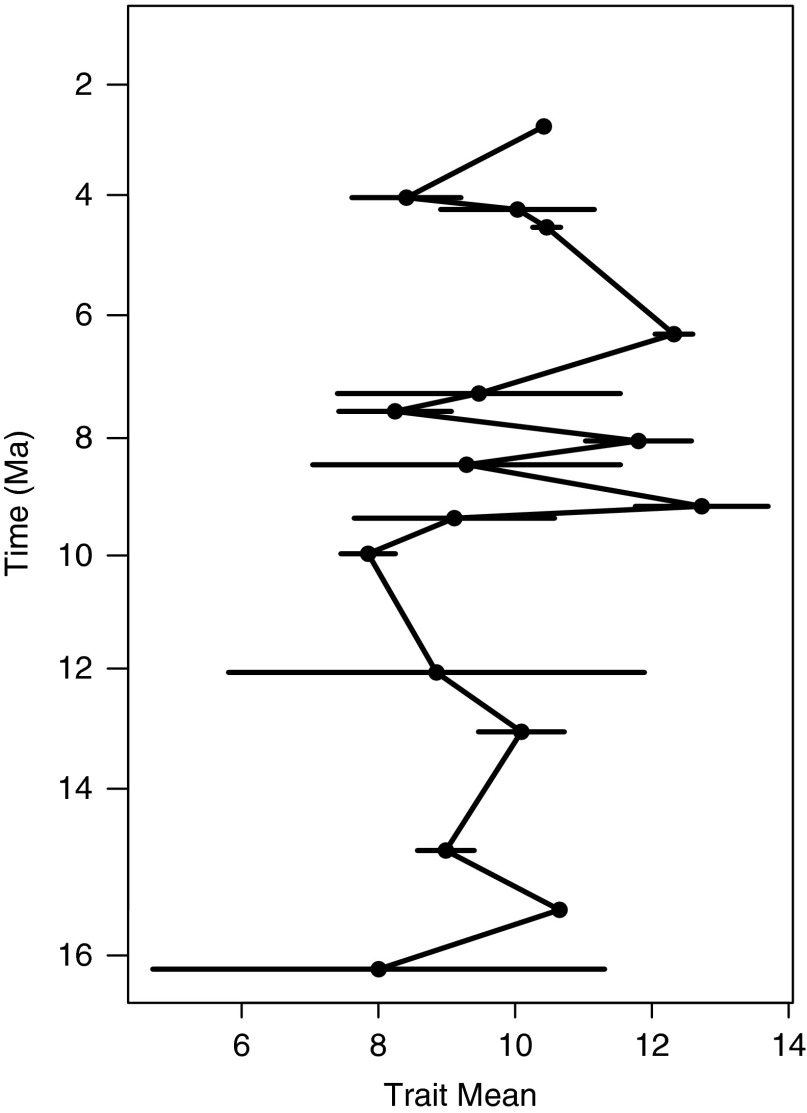
Evolutionary trajectory of *Carcharocles megalodon* body size. Bars represent standard errors of the mean.

[H1: *Carcharocles megalodon* increased in size through time, reaching its largest size prior to extinction]. When testing for the three models of trait evolution, we found that stasis is the one that best fits our data, accounting for 97% of the Akaike weight and greatly outperforming the UWR and GWR models (Table 3). This trend is supported even when using raw data (Supplementary Table S3, Supplementary Fig. S3). We therefore reject our hypothesis of body-size increase through time. This result contrasts with the size increase trend seen in the megatooth lineage (Fig. 5).

**Table 3:** Model-fitting results for *Carcharocles megalodon* body size trends. Largest Akaike weight (best fit) in bold.

	logL	AICc	Akaike weight
GRW	−36.22	77.37	0.004
URW	−36.36	75.00	0.016
Stasis	−30.80	66.53	**0.981**

Stasis in body size was previously proposed for *C. megalodon* on the basis of dental measurements (Pimiento et al. 2010). However, because the aim of that work was to compare tooth measurements (not body size) from a particular area (nursery), the comparisons were made using only three localities, based on a limited sample size, and not statistically tested. Conversely, here we used rigorous quantitative methods (i.e., Hunt 2006, 2008; Hunt and Carrano 2010) to test for different hypotheses of mode of trait body-size evolution.

Although stasis has been widely studied, no consensus has been reached on the causal mechanisms (Estes and Arnold 2007; Hunt 2007; Hunt and Rabosky 2014). It has been proposed that stasis could be caused by stabilizing natural selection, genetic and environmental constraints, resource competition, habitat selection, and/or geographic structure, among others (Eldredge et al. 2005; Estes and Arnold 2007; Hunt 2007; Hunt and Rabosky 2014). From these, stabilizing selection and geographic structure are particularly supported (Hunt 2007). Stabilizing selection causes a species’ size to be relatively close to its optimum (Kozlowski and Gawelczyk 2002) and when this optimum does not change much over time, stasis is observed. Similarly, the geographic range of a widespread species can cause stasis due to spatially heterogeneous natural selection acting across semi-isolated populations (Eldredge et al. 2005; Hunt 2007; Hunt and Rabosky 2014). Accordingly, stasis is common when a taxon has widespread distributions, lives in variable environments, and is insensitive to environmental fluctuations (Sheldon 1996; Benton and Pearson 2001). Because *C. megalodon* body size is both invariant in terms of size-frequency distributions (keeping a relatively constant modal [optimum?] value) and variant across hemispheres and oceans over geologic time, stabilizing selection and/or geographic structure may be (either mutually or exclusively) the mechanisms causing stasis in this species.

### Broader Implications

To our knowledge, body-size trends of large predatory sharks have never been studied before over geologic time. Our results have three broader implications that provide a deep-time perspective to the understanding of the body-size trends of marine apex predators:

1. The left-skewed distribution of *C. megalodon* body size, both in the total temporal range and throughout the different periods studied, suggests a selective pressure favoring larger individuals. At ecological scales, and despite body-form similarities between large and small predatory sharks (Irschick and Hammerschlag 2014), larger individuals tend to prey upon larger animals (Lucifora et al. 2009). This trend is related to an ontogenetic dietary shift whereby smaller individuals avoid large (possibly dangerous) prey, whereas larger individuals consume a broader range of prey sizes (Lucifora et al. 2009; Estrada et al. 2006). This pattern has also been observed across different species of terrestrial predators (Peters 1983; Carbone et al. 1999). The left-skewed distribution of *C. megalodon* body size may therefore be the result of a long-term selective pressure on marine predatory sharks that favors consumption of a broader range of prey, increasing their impact in the structure of food webs (e.g., Steneck 2013).
2. Given the widespread distribution of a large cosmopolitan apex predator such as *C. megalodon*, the body-size variations found across oceans and hemispheres may be a result of the heterogeneous ecological conditions that they faced. Currently, sympatric populations of cosmopolitan predatory marine mammals such as the killer whale (*Orcinus orca*) are genetically distinguishable. This might be a result of assortative mating, which eventually produces morphological (e.g., body size) and behavioral differences between populations through generations (Hoelzel and Dover 1961). Similarly, the great white shark (*Carcharodon carcharias*) has demographically isolated populations due to their high degree of site fidelity (Jorgensen et al. 2009). Our study of *C. megalodon* body-size trends through space and geologic time suggests that the ecological distinctiveness of geographically discrete populations of large cosmopolitan marine apex predators may shape their body-size trends in deep time.
3. Finally, the lack of size change in *C. megalodon* throughout geologic time contrasts with the size increase trend observed not only in the megatooth lineage but also in other lineages of marine predators such as toothed whales (Odontoceti) (Pyenson and Sponberg 2011). Given that sharks have slower evolutionary rates than mammals (Martin et al. 1992), the lack of body-size change in *C. megalodon* may be the result of the inherent characteristics of shark species, which potentially make them particularly resilient to environmental changes (Martin et al. 1992; Pimiento et al. 2013a). A disconnection between micro- and macroevolutionary body-size patterns (i.e., stasis in the species vs. size increase in the lineage) could be an evolutionary consequence of such characteristics. The macroevolutionary mechanisms that produce the body-size increase in lineages of large marine predators are the subject of a future investigation.

## Conclusions

We found that *Carcharocles megalodon* body size had a left-skewed distribution and was significantly different between hemispheres and ocean basins through geologic time. In addition, we found stasis as the mode of size evolution of *C. megalodon*, and thus reject our hypothesis of body-size increase over geologic time. Given that *C. megalodon* is a long-lived giant predator with a fossil record of ~14 Myr, it represents an excellent study system to provide a deep-time perspective to the understanding of body-size trends of marine apex predators. For instance, this study suggests that (1) a selective pressure in predatory sharks for consuming a broader range of prey may favor larger individuals and produce left-skewed distributions over geologic time, (2) body-size variations in cosmopolitan large apex predators may depend on the predators’ interactions within geographically discrete communities, and (3) the inherent characteristics of shark species can produce a lack of net size changes over geologic time, even though the species’ lineage shows size increase. Future research on body-size patterns of additional large apex predators (e.g., other megatooth sharks, toothed whales, plesiosaurs, mosasaurs, archaeocetes) would allow a more complete understanding of the macroevolutionary mechanisms that produce body-size increases, the evolution of gigantism, and the role of body size in extinction risk.

## Supplementary material

*Supplemental materials deposited a* *t Dryad*: doi:10.5061/dryad.6q5t4
